# Assessment of Cellular Estrogenic Activity Based on Estrogen Receptor-Mediated Reduction of Soluble-Form Catechol-*O*-Methyltransferase (COMT) Expression in an ELISA-Based System

**DOI:** 10.1371/journal.pone.0074065

**Published:** 2013-09-06

**Authors:** Philip Wing-Lok Ho, Zero Ho-Man Tse, Hui-Fang Liu, Song Lu, Jessica Wing-Man Ho, Michelle Hiu-Wai Kung, David Boyer Ramsden, Shu-Leong Ho

**Affiliations:** 1 Division of Neurology, Department of Medicine, University of Hong Kong, Hong Kong SAR, China; 2 School of Medicine and School of Biosciences, University of Birmingham, Birmingham, United Kingdom; 3 Research Centre of Heart, Brain, Hormone and Healthy Aging, University of Hong Kong, Hong Kong SAR, China; Kaohsiung Chang Gung Memorial Hospital, Taiwan

## Abstract

Xenoestrogens are either natural or synthetic compounds that mimic the effects of endogenous estrogen. These compounds, such as bisphenol-A (BPA), and phthalates, are commonly found in plastic wares. Exposure to these compounds poses major risk to human health because of the potential to cause endocrine disruption. There is huge demand for a wide range of chemicals to be assessed for such potential for the sake of public health. Classical *in vivo* assays for endocrine disruption are comprehensive but time-consuming and require sacrifice of experimental animals. Simple preliminary *in vitro* screening assays can reduce the time and expense involved. We previously demonstrated that catechol-O-methyltransferase (COMT) is transcriptionally regulated by estrogen via estrogen receptor (ER). Therefore, detecting corresponding changes of COMT expression in estrogen-responsive cells may be a useful method to estimate estrogenic effects of various compounds. We developed a novel cell-based ELISA to evaluate cellular response to estrogenicity by reduction of soluble-COMT expression in ER-positive MCF-7 cells exposed to estrogenic compounds. In contrast to various existing methods that only detect bioactivity, this method elucidates direct physiological effect in a living cell in response to a compound. We validated our assay using three well-characterized estrogenic plasticizers - BPA, benzyl butyl phthalate (BBP), and di-n-butyl phthalate (DBP). Cells were exposed to either these plasticizers or 17β-estradiol (E2) in estrogen-depleted medium with or without an ER-antagonist, ICI 182,780, and COMT expression assayed. Exposure to each of these plasticizers (10^-9^-10^-7^M) dose-dependently reduced COMT expression (p<0.05), which was blocked by ICI 182,780. Reduction of COMT expression was readily detectable in cells exposed to picomolar level of E2, comparable to other *in vitro* assays of similar sensitivity. To satisfy the demand for *in vitro* assays targeting different cellular components, a cell-based COMT assay provides useful initial screening to supplement the current assessments of xenoestrogens for potential estrogenic activity.

## Introduction

Estrogens regulate endocrine, developmental and reproductive systems in human. Many chemicals or substances, both synthetic and naturally occurring, mimic or inhibit the actions of estrogen (generally classified as xenoestrogens). If introduced in humans, such chemicals disrupt the normal actions of estradiol and cause a wide spectrum of endocrine disruptive problems at all stages of life, including early puberty, disturbance of spermatogenesis and hormone synthesis, and prostatic and breast cancers [1,2]. Catechol-O-methyltransferase (COMT) is an enzyme ubiquitously expressed in various human tissues to degrade catecholamines and thus facilitate the removal of hormones (e.g. estrogen) from the body. The carcinogenic intermediate catechol estrogens (e.g., 4-hydroxyestradiol) generated from hydroxylation of endogenous estrogens and xenoestrogens by CYP450 are inactivated by COMT, or they are further oxidized into reactive quinones causing oxidative stress [3]. Endogenous COMT activity is a major determinant in facilitating estrogen metabolism, and it plays an important role in the pathophysiology of different human disorders including Parkinson’s disease, depression, schizophrenia, hypertension, and various estrogen-induced cancers [3–9].

To address the health concerns of the endocrine disruptors, large number of chemicals are required to be tested for estrogenic endocrine disrupting properties. The classical method of carrying out such an assessment is the *in vivo* “two-generation” rat test [10]. However, this requires a considerable number of animals, which runs counter to the reduction in the number of animals used in animal testing protocols. Subsequently, several means of speeding up the testing process and reducing the number of mammals required have been devised. These include the *in vivo* luciferase-dependent gene promoter activity tests in zebrafish [11], and a series of *in vitro* screening assays which focus on three different aspects: the estrogenic-stimulation to cell proliferation assay (E-SCREEN), the estrogen receptor binding assay, and the estrogen-receptor (ER) transcriptional activation via reporter gene in yeast or mammalian cells [12,13]. These assays have advantages and limitations [14,15]. Nevertheless, there is no single assay that can satisfactorily predict such a diverse spectrum of estrogenic effects, nor was it be able to determine the adverse physiological responses induced by the testing agents.

Previously we have shown that endogenous COMT expression is not only transcriptionally regulated by estrogen via the estrogen-response-element (ERE) in its gene promoter region [8,16] but also by compounds of widely different chemical structures but which have the capacity to either mimic or antagonize the actions of estradiol [17,18]. In particular, xenoestrogens such as polychlorinated biphenyls (PCBs) and phthalate-based plasticizers (e.g. bis-isohexyl phthalate, octylphenol), which are commonly used in the manufacture of plastic products, have long been classified as potent estrogenic endocrine disruptors. Thus assaying changes of COMT expression in response to these estrogenic agents may be one of the useful methods to estimate estrogenicity. In this communication, we describe a cell-based system to assess estrogenic agents, which utilizes a novel competitive ELISA, based on exclusive expression of soluble-form catechol-O-methyltransferase (S-COMT) isoform in a ER-positive human breast adenocarcinoma MCF-7 cell line [19]. To evaluate the assessment of estrogenic effects by COMT assay, we have tested three common plasticizers - bisphenol-A (BPA), benzyl butyl phthalate (BBP) and di-n-butyl phthalate (DBP), with known disruptive properties to estrogenic action [20]. The results from this novel assay were compared with those obtained using classical SDS-PAGE/Western blotting.

## Results

### BPA, BBP and DBP dose-dependently decreased COMT protein expression in Western blotting

**Figure 1:**
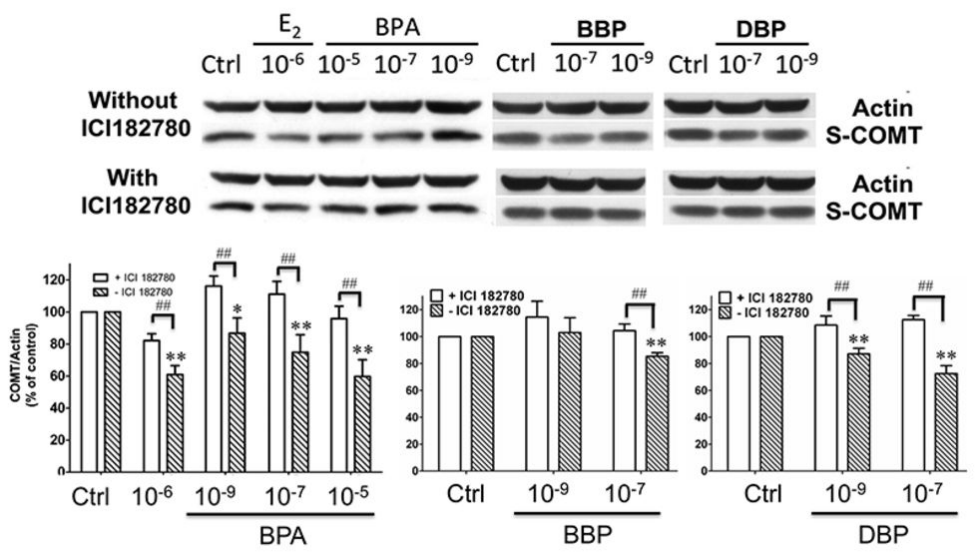
Three common plasticizers (bisphenol-A (BPA), benzyl butyl phthalate (BBP), and di-n-butyl phthalate (DBP)) dose-dependently decreased COMT expression via estrogen receptor (ER) in MCF-7 cells. Cells were treated with graded doses of each of the three compounds, in the absence or presence of ICI 182,780 (ER antagonist). The resultant changes of soluble-COMT (S-COMT; 23kDa) protein expression were determined by Western blotting. Equal loading of samples were normalized by actin (42kDa). Results are mean ± SEM of four separate experiments (n=4). Statistical significance at the level of *p<0.05 or **p<0.01, as compared to untreated controls. ^##^ p<0.01, as compared between the two designated treatment groups.

When MCF-7 cells were cultured with graded doses of either BPA (10^-5^, 10^-7^, and 10^-9^M), or BBP (10^-7^ and 10^-9^M), or DBP (10^-7^ and 10^-9^M), for 48 hours in charcoal-stripped medium, there was a concentration dependent decrease in S-COMT levels for all three tested compounds relative to controls (BPA: 10^-5^M: p<0.01; 10^-7^M: p<0.05, n=4; BBP: 10^-7^M: p<0.01, 10^-9^M: p<0.05 n=3; DBP: 10^-7^M: p<0.01; n=3) (Figure 1). However, when these cells were treated together with the ER-antagonist, ICI 182,780, such concentration dependent decrease of COMT expression was abolished (p<0.05), as compared with the corresponding group without ICI 182,780 (Figure 1). The final concentration of solvent DMSO in culture medium was 0.05% (v/v), which did not affect COMT protein expression level (data not shown).

### Development of competitive COMT ELISA - Structure of human recombinant MB-COMT-NE standard protein and validation of NE antibody specificity

**Figure 2:**
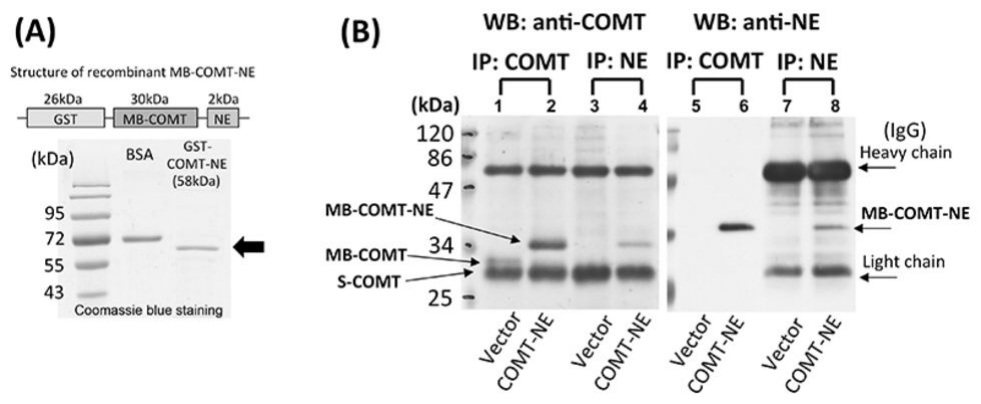
Bacterial expression, purification, and characterization of recombinant MB-COMT protein standard. (A) Structure of the engineered gene to yield full-length human recombinant membrane-bound-COMT (MB-COMT) protein, and the purity of the recombinant protein by SDS-PAGE following extraction from *E. Coli*. NE is a novel 18-amino-acid protein tag attached to MB-COMT for detection in the competitive COMT ELISA. (B) Results of SDS-PAGE/Western blotting probed with anti-COMT (lanes 1-4) or anti-NE (lanes 5-8) following immunoprecipitation by either anti-COMT (lanes 1, 2 & 5, 6) or anti-NE (lanes 3, 4 & 7, 8).

The protein structure of the recombinant MB-COMT and its purity after extraction from *E. Coli* is shown in SDS-PAGE after Coomassie blue staining of the gel (Figure 2A). These proteins extracted from *E. Coli* were used as standard proteins in the ELISA. On the other hand, the specificity of NE antibody was validated by immunoprecipitation and SDS-PAGE/Western blot in HEK293 cell lysate overexpressing MB-COMT-NE (Figure 2B). Recombinant MB-COMT-NE protein in total cell lysate extracted from HEK293 cells was immunoadsorbed by either anti-COMT or anti-NE antibodies, and cross-detected on SDS-PAGE/Western blots. On the Western blots of lysate which has undergone immuno-precipitated with anti-COMT and probed with either anti-COMT or anti-NE antibodies, there was no antibody binding at a position appropriate for MB-COMT in vector control cell lysate (i.e. without expression of MB-COMT-NE (Figure 2B, lane 1 and 3). In contrast, single band was observed corresponding to MB-COMT-NE (32kDa) in anti-COMT pull-down lysate from cells overexpressing MB-COMT-NE in blots probed with either anti-COMT or anti-NE (Figure 2B, lanes 2 and 4). Similar results were seen when cell lysates were immuno-precipitated with anti-NE and the resultant precipitated subjected to SDS-PAGE/Western blotting (Figure 2B, lanes 5 to 8).

### Characterization of COMT ELISA

**Figure 3:**
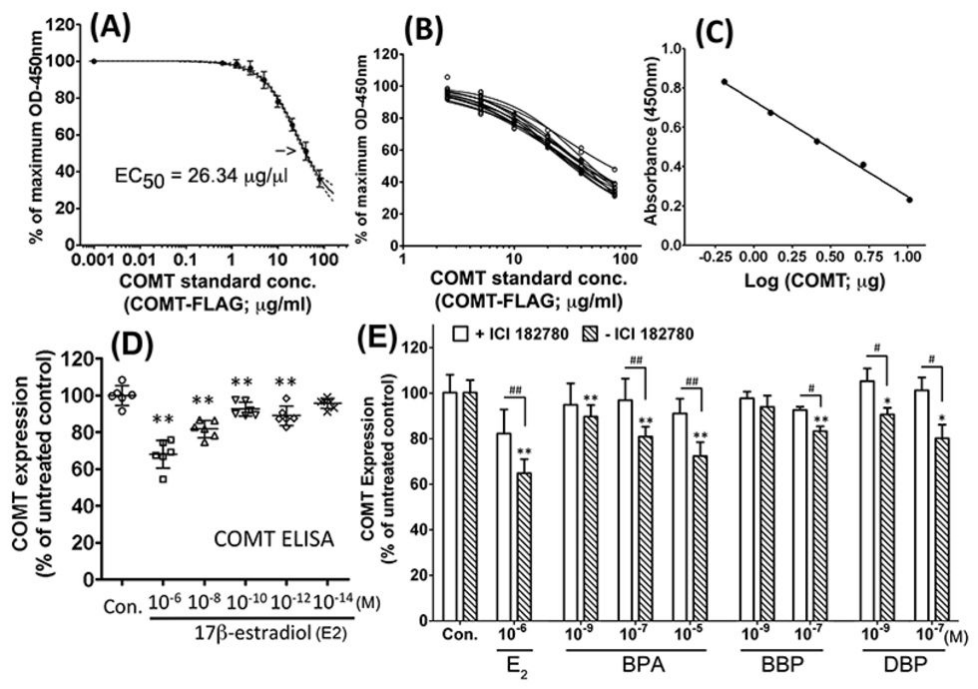
Characterization and application of COMT ELISA. (A) EC_50_ determination from mean competitive curve ± S.E.M. (B) Combination plots of 13 individual standard curves illustrating the consistency of the method. (C) Calibration curve used in the ELISA generated from a serial dilution of untreated MCF-7 lysate (COMT concentration: 36.4µg/mg total protein). The lysate gave a linear response under the conditions of the ELISA in a highly similar fashion to that of the purified recombinant MB-COMT standards. (D) Effects of graded doses of 17β-estradiol (E2) on COMT expression in MCF-7 cells determined by COMT ELISA after 48-hour treatment. (E) Effects of treatment of MCF-7 cells with graded doses of BPA, BBP, and DBP, in the presence or absence of ICI 182,780 on COMT expression determined by COMT ELISA. Results are mean ± SEM of four separate experiments (n=4). Statistical significance at the level of **p<0.01, as compared to untreated controls. ^##^ represents significance at level of p<0.01, ^#^ p<0.05, between the two designated treatment groups.

The competitive ELISA gave responses which were inversely correlated to standard recombinant MB-COMT concentration and when fitted by a nonlinear curve-fitting algorithm gave sigmoid curves with r^2^ values ranging between 0.97 and 1.0, determined as a plot of log agonist concentration against response in six independent trials; the mean curve ± S.E.M. with mean EC_50_ (26.34µg/ml; 95% confidence intervals = 20.26 to 34.2µg/ml) is shown in Figure 3A. The above six curves were plotted with a further seven curves (response at x mg MB-COMT concentration divided by response at zero MB-COMT concentration against log MB-COMT concentration) to illustrate the consistency of the method (Figure 3B), with ±4% standard error for inter-variation. Intra-variation between eight wells assayed for the same sample was ±1.5%. These gave curvilinear plots with r-values ranging between 0.97 and 1.0. The average sensitivity (minimum concentration of MB-COMT practically detectable) of the ELISA was 0.8ng/µl.

It was important to determine whether the recombinant protein used as standards in the ELISA and human soluble-COMT (S-COMT) in MCF-7 cell lysate behaved in the same way in the ELISA. To demonstrate this, MCF-7 lysate was serially diluted with BSA solution to maintain an equal total protein concentration in all samples. Aliquots of these replaced the recombinant standards within the ELISA. The diluted lysates gave a linear response in the ELISA a similar manner to the recombinant standards (Figure 3C). The concentration of COMT in lysates of normal untreated MCF-7 cells was at a level of 36.4µg/mg total protein, being comparable with results in previous literature [21] (i.e. 8.1 ± 1.5 µg/mg).

### COMT assay by competitive ELISA – Effect of 17β-estradiol

Treatment with 17β-estradiol (10^-12^-10^-6^M) for 48 hours caused a reduction in COMT expression compared with the results for untreated control cells (10^-12^-10^-6^M, all p<0.01, n=6; Figure 3D) in a dose-dependent manner (Spearman r = 0.81; p<0.0001, n=30). Such reduction can be effectively blocked by ER antagonist, ICI 182,780, confirming that reduction of COMT by 17β-estradiol is mediated by ER (Figure 3E).

### Suppression of COMT expression by BPA, BBP and DBP in COMT ELISA was comparable to western blotting results

Using aliquots of the same samples after treated with different concentrations of BPA, BBP, DBP or 17β-estradiol, the resultant changes in COMT protein levels were determined in COMT ELISA and SDS-PAGE/Western analysis. Exposure to BPA (10^-9^-10^-7^M) reduced COMT expression in a dose-dependent manner (Spearman r = 0.83; p<0.0001, n=18) in six separate estimations in duplicate. Furthermore, BBP (10^-7^M) and DBP exposure (10^-9^-10^-7^M) significant reduced COMT expression (all p<0.05), which was significantly attenuated by ICI 182,780 (all p<0.05) (Figure 3E). The accuracy of the ELISA was illustrated by the fact that the average coefficient of variation of the duplicates of the COMT estimates following BPA treatment over the 6 curves was 7%. This suppressive effect of all three plasticizers, BPA, BBP and DBP, was abolished by the presence of ICI 182,780 (Figure 3E). The estrogenic effect of these plasticizers as expressed by percentage reduction in COMT expression from ELISA was similar and comparable to those determined by SDS-PAGE/Western analysis. For the two methods, the Spearman rank correlation coefficient is r=0.78 (p<0.0001, n=24).

## Discussion

Environmental contaminants with endocrine disrupting potential pose an increasing threat to public health. Current methods of assessing such compounds involve a combination of *in vivo* and *in vitro* assays, including the comprehensive “two-generation” rat test [10]. However, these *in vivo* tests are always time-consuming and expensive that it would be virtually impossible to assess all chemicals in short period of time; even the “one-generation test” [22] would require a formidable number of animals and time to complete the required list in the near future. Alternatively, there are *in vitro* screening assays for assessment of estrogenic and hormonal activity [12,13]. However, due to the nature of diverse variety of testing substances targeting different cellular components, detection by one single assay would not adequately cover all potential cellular toxicity pathways. Given the inherited limitations for the existing *in vitro* assays [14,15], combination of *in vivo* and *in vitro* assays remains by far the best strategy.

One possible strategy in developing a more representative screening would be to target the physiological response of various endogenous protein markers, as a reflection of downstream convergence from various estrogenic signals. In this study, we described an *in vitro* assay to quantify estrogenic effects based on changes of cellular COMT protein level. This novel bioassay is based on the physiological response of MCF-7 cells to suppress S-COMT protein expression under ER-mediated estrogenic stimulation [8,16]. Both endogenous estrogen and its catechol metabolites can suppress COMT expression and activity, and has been suggested to be a risk factor for estrogen-associated cancers [21]. Moreover, COMT expression is suppressed by xenoestrogens, including phthalate-based plasticizers [17,18]. It is not surprising that absorption of these estrogenic agents may adversely affect COMT expression and perturb the metabolic processes of endogenous estrogen. Therefore, this assay not only determines a direct transcriptional regulatory effect of COMT via estrogenic activity of a compound, but also implicates potential adverse effects to estrogen metabolism due to abnormal suppression of COMT expression.

Human COMT is retrospectively regulated by estrogen [8,16]. The 5’ flanking region of the COMT gene that gives rise to the S-COMT transcript contains numerous well-separated estrogen-response-element (ERE) half-sites, which are involved in the estrogenic regulation of S-COMT expression [8]. These differ from classical EREs in that the number of bases between the half-sites is much greater than between half-sites that form classical EREs. In addition, S-COMT is down-regulated by estradiol, and so effects on S-COMT via these sites are likely to involve different mechanisms of interaction of the ER with DNA and different co-factors than those mediating gene transcription via classical EREs. Thus, the results may reflect alternative mechanisms that supplement results of tests based on the classical ERE to detect potential estrogenic endocrine disruptors. As shown by our ELISA assay at graded concentrations of estradiol (Figure 3D), the sensitivity of response in the form of suppression of COMT protein expression in MCF-7 cells can be achieved at the lowest estradiol concentration of picomolar (10^-12^M) level. This sensitivity level is comparable with the detection limit as reported for the traditional estrogenic cell proliferation assay (i.e. ~30pM) [14]. Although the mammalian cell (e.g. T47D human breast cancer cells) reporter gene assay offers even higher assay sensitivity up to femtomolar (10^-15^M) level, these assays require utilization of transfected reporter gene construct engineered with multiple EREs. Due to the amplification of response signal by the multiple response elements and the ectopic expression of luciferase, this way of detection greatly increases the assay sensitivity, but may not satisfactorily reflect the actual physiological situation. For example, previous studies have shown that BPA binds with low affinity to the ER but exerts serious developmental problems *in vivo* [23].

To demonstrate the efficacy of our assay, we investigated the estrogenic effects of three plasticizers commonly used in plastics manufacturing. Chronic exposure of BPA, similar to other phthalate-based plasticizers, e.g. BBP and DBP, is widely recognized as environmental pollutant and a major risk factor to hormone-related carcinogenesis in rodents [24–26]. In this test system, we have shown that all three tested plasticizers can significantly reduced S-COMT level via ER-mediated transcription in a dose-dependent manner, thus illustrating the possibility that different arrangement of the ER half-sites and different co-factors may well affect ligand binding to the receptor and hence the need for a range of simple and sensitive assays to ensure adequate assessment of estrogenic potential in an untested compound. The specificity of estrogenic action was shown by the fact that ER antagonist, ICI 182,780, blocked the action of these plasticizers in suppressing COMT protein expression. Since the traditional ER binding and the transcriptional activation assays have limitations to differentiate specific pathways affected by the tested compounds, a combination of a physiologically sensitive marker (S-COMT expression) and a simple immuno-assay for the marker (the novel competitive ELISA) makes the system suitable as a preliminary screen for potential estrogenic agents. We verified the results of our novel ELISA by showing that these were highly correlated with those obtained by traditional Western blotting. The applicability of this assay is further supported by our previous studies that a variety of phthalate-based plasticizers, e.g. DEHP, DINP [17], and polychlorinated biphenyls [18] can modulate S-COMT expression in MCF-7 cells. Therefore, the relative changes in COMT expression after exposure to potential estrogenic compounds may be correlated to the disruption, which may potentially occur to estrogen metabolism via ER-mediated COMT suppression.

In conclusion, our study demonstrates a quick assay of estrogenic effects in a cell-based ELISA, as validated by suppression of COMT expression by three estrogenic plasticizers, BPA, BBP, and DBP. In response to the increasing demand in screening methods for endocrine disrupting potential, assessment of cellular response of COMT expression to estrogenic agents serves to screen hazardous chemicals in a fast and economical manner, complementing current *in vitro* and *in vivo* tests for estrogenic activity.

## Materials and Methods

### Materials

All test reagents and chemicals including the tested plasticizers (i.e. bisphenol-A (BPA), benzyl butyl phthalate (BBP), and Di-n-butyl phthalate (DBP)), dithiothreitol (DTT), 17β-estradiol (E2), and phenylmethylsulfonyl fluoride were purchased from Sigma-Aldrich Chemical Company, (Milwaukee, WI, USA). Human breast adenocarcinoma MCF-7 cell line (human breast adenocarcinoma; HTB-22), human embryonic kidney HEK293 cell line (CRL-1573), and human neuroblastoma SH-SY5Y cells (CRL-2266; ATCC) were obtained from the American Type Culture Collection (ATCC; Rockville, MD). Charcoal-stripped bovine calf serum, RPMI without phenol red indicator, penicillin-streptomycin, insulin and 10% sodium dodecyl sulfate (SDS) stock solution were purchased from GIBCO/BRL (Grand Island, NY, USA). Prestained protein molecular weight standard markers were from Fermentas Int. Inc. (Ontario, Canada). Horseradish peroxidase (HRP)-conjugated anti-sheep immunoglobulin antiserum was obtained from DAKO (Glostrup, Denmark). ECL-plus western blotting detection reagents were purchased from Amersham Pharmacia Biotech (NJ, USA). The estrogen receptor antagonist (ICI 182,780) was purchased from Tocris (Ellisville, MO, USA). Sheep polyclonal anti-human COMT antibody was raised by The Binding Site Ltd. (Birmingham, UK), and affinity purified as previously described [14]. This antibody is directed against a linear epitope of human COMT peptide which is present in both membrane bound (MB)- and soluble (S)- forms of COMT. The mouse monoclonal anti-human actin (C-2) was purchased from Santa Cruz Biotechnology (Santa Cruz, CA, USA).

### Cell culture and treatments

Human MCF-7 cells were chosen as a cell model for assessment of estrogenic effects because these cells are responsive to estrogen via classical estrogen receptor (ER)-mediated pathway [17]. The estrogen-responsive S-COMT isoform is dominantly expressed in MCF-7 cells [14]. MCF-7 cells in RPMI containing 10% heat-inactivated fetal bovine serum, antibiotics (100U/ml of penicillin and 100µg/ml of streptomycin), and 1µg/ml insulin in humidified 5% CO_2_ at 37°C. Cells (10^5^) were seeded onto 24-well plate and incubated in fresh RPMI. Both 17β-estradiol (a potent S-COMT suppressor) was dissolved at stock concentration of 1mM in PBS. The three testing compounds, BPA, BBP and DBP were freshly prepared at stock concentration of 10mM in DMSO. After the cells had reached 70% confluence, the medium was replaced with phenol red-free RPMI supplemented with 10% charcoal-stripped calf serum depleted of exogenous estrogen, antibiotics and insulin, for 48 hours. After the pre-incubation, cells were exposed to either a fixed concentration of E2 (10^-6^M; positive control) or graded concentrations of BPA (10^-5^, 10^-7^, and 10^-9^M), BBP (10^-7^ and 10^-9^M), or DBP (10^-7^ and 10^-9^M), with or without the anti-estrogen, ICI 182,780 (10^-7^M). Cells were harvested after an additional 48 hours of incubation for determination of COMT expression level by ELISA, and SDS-PAGE/Western analysis as described below. Experiments were repeated a minimum of three times.

### Development of competitive ELISA for COMT

Human COMT is expressed in two forms originating from transcripts of the same gene, which has two transcription initiation sites – S-COMT in cytosol and membrane-bound COMT (MB-COMT). The development of a typical competitive ELISA requires sources of COMT for the standard control protein to generate the standard curve, and a tagged COMT to act as a detectable competitor against either the standard controls or endogenous COMT in samples. Both S-COMT and MB-COMT can be captured by anti-COMT detection antibody in the ELISA. For simplicity of cloning, only the full-length MB-COMT was chosen as the basis of the detectable competitor, which was tagged with a novel epitope – termed “NE” (*patent application -US 20100081147; EP 2328909; CN 102203119*), whereas the standard control MB-COMT protein was tagged with FLAG^TM^ to aid detection in the purification steps following synthesis. These two recombinant proteins compete with each other at different ratio for the binding to anti-COMT detection antibody in the ELISA to generate the standard curve. An antibody against the NE tag was required as part of the detection step in the ELISA.

### Molecular cloning of recombinant MB-COMT-NE protein

A full length human MB-COMT cDNA was generated from total RNA isolated from human neuroblastoma SH-SY5Y cells (CRL-2266; ATCC) by one-step RT-PCR (Titan One Tube RT-PCR Kit, Roche) according to the manufacturer’s protocol, using a pair of primer specifically designed for addition of NE cDNA fragment at 3’-end of MB-COMT. To generate BamHI-COMT-NE-EcoRI cDNA fragment for directional cloning into the expression plasmid (pcDNA3.1(+); Invitrogen or pGEX-6p1; GE Healthcare), restricted enzyme sites (BamHI and EcoRI) were inserted by a second PCR using forward primer for MB-COMT (5’-CGCGGATCCGCCACCATGCCGGAGGCCCCGCCTCTGC-3’); and reverse primer containing the NE sequence and EcoRI restriction site (5’-CTGGAATTCTCAGCTTTCGTTATCATCATAGCTTTCTTCCTGGTTGCTACGCGGGTTTTCTTTGGTGGGCCCTGCTTCGCTGCCTGGGC-3’). The MB-COMT DNA insert (BamHI-COMT-NE-EcoRI) and empty vector (pcDNA3.1(+) or pGEX-6p1) were digested separately by restriction enzymes (BamHI and EcoRI), and ligated to the pcDNA3.1(+) or pGEX-6p1 vector at 4^o^C overnight. A similar strategy was used to generate MB-COMT-Flag, where the NE sequence was replaced with the FLAG sequence.

### Expression of recombinant MB-COMT-NE and MB-COMT-FLAG proteins

For expression of either recombinant MB-COMT-NE or MB-COMT-FLAG protein in *E. Coli* (BL21), a positive clone was culture in LB broth supplemented with ampicillin (50g/ml) and incubated at 37°C with vigorous shaking until the optical density reading of the bacterial culture reached ~0.6 (at λ = 600nm). The culture was induced with isopropylthio-β-D-galactoside (0.1mM) and cultured for another 4 hours. Cells were collected by centrifugation and were sonicated in ice-cold lysis buffer (50mM Tris-Cl, pH 7.5, 200mM NaCl), supplemented with 5mM 1,4-dithiothreitol (DTT) and 1mM PMSF. The expressed recombinant MB-COMT-NE or MB-COMT-FLAG protein from the bacterial lysate was purified by passing through a commercially available glutathione-S-transferase (GST)-purification column (GSTrap™ FF, GE Healthcare). The target protein band was visualized by SDS-PAGE after Coomassie blue staining of the gel, and characterized by Western blotting.

### Construction of COMT competitive ELISA

Ninety-six-well plates were pre-coated with varying amounts of a polyclonal COMT antibody directed against a common epitope in S- and MB-COMT (capture antibody) at 4°C overnight. A series of diluted MB-COMT-NE standards were added to the wells and incubated at 37°C for 2 hours to determine the optimum amount of capture antibody required. After subsequent washes with TBS-Tween, rabbit anti-NE antibody (detection antibody) and HRP-conjugated anti-rabbit antibody were sequentially dispensed to assay wells and incubated at room temperature for 1 hour. After washes, TMB substrate was added and incubated in the dark for 30 min for colorimetric development. The optical density (OD) of each well at 450nm was measured using a microplate reader immediately after addition of sulphuric acid (0.1M, 1: 1 v/v). The optimum amount of capture antibody was 0.1µg per well. Once this was established, a series of standard curves was developed to ascertain the optimum amount of MB-COMT-NE. Once the optimum coating with capture antibody and amount of competitor MB-COMT-NE were determined, a series of standard curves was developed using a range of MB-COMT-FLAG concentrations (0-80µg) to ascertain the sensitivity and reproducibility of the assay.

### Western blotting

Changes of S-COMT expression in MCF-7 cells were determined by SDS-PAGE/western blotting following our previously described protocol [17] to compare the results with those obtained by our COMT ELISA. Briefly, cells were washed with ice-cooled PBS and harvested in buffer containing: 50mM Tris-Cl, pH 7.4, 150mM NaCl, 1% sodium deoxycholate, 1mM EDTA, 1% Triton X-100, 0.1% SDS, 1mM phenylmethylsulfonyl fluoride, supplemented with protease inhibitor cocktail at 4°C. Lysates were clarified by centrifugation at 4°C for 15 min. at 12,000 r.p.m. Sample protein concentrations were determined by Bradford assay. Equal amounts of protein were boiled for 10 min at 95^o^C in sample buffer containing: 62.5mM Tris, pH 6.8, 100mM DTT, 2% SDS, 10% glycerol, and 0.002% bromophenol blue. Samples (20µg per lane) were electrophoresed on 12% SDS-polyacrylamide gels and then transferred onto nitrocellulose membrane. Resulting blot were blocked with 5% non-fat skimmed milk in TBS and probed with antibodies directly against COMT (1:1000) or β-actin (1:1000). For chemiluminescence detection, blots were incubated with HRP-conjugated secondary antibodies (1:5000) followed by ECL-plus substrate detection. Immunoblots were quantified by computerized scanning densitometry.

### Effect of 17β-estradiol and BPA on S-COMT expression in competitive ELISA

MCF-7 cell lysates were prepared from cells treated either with graded doses of E2, BPA, BBP or DBP as indicated above and normalized to the same concentration with ice-cooled PBS supplemented with protease inhibitor cocktail (Roche). Equal amounts of lysate (50µg) were mixed with 2µg of recombinant MB-COMT-NE and topped up to 100µl with BSA-containing sample diluent. A serial dilution of standards each containing recombinant MB-COMT-NE (2µg) and MB-COMT-FLAG (0-80µg) in 100µl sample diluent were prepared. Duplicate aliquots (100µl) of either standards or cell lysate preparations were added to wells and the ELISA performed as above.

### Statistical analysis

Statistical analyses were carried out using the Prism (GraphPad Software Inc.). Data are expressed as mean ± standard error mean (S.E.M.) from at least three independent experiments. Statistical significance among individual groups was calculated by the one-way ANOVA followed by Tukey’s *post-hoc* multiple comparison, and independent *t*-test. Spearman correlation coefficients (r) between various parameters were calculated, because normal distributions could not be assumed. Differences and r values were considered significant at a level of p<0.05.

## References

[1] Waring RH, Ayers S, Gescher AJ, Glatt HR, Meinl W et al. (2008) Phytoestrogens and xenoestrogens: the contribution of diet and environment to endocrine disruption. J Steroid Biochem Mol Biol 108(3-5): 213-220. doi:10.1016/j.jsbmb.2007.09.007. PubMed: 17933522. 17933522 10.1016/j.jsbmb.2007.09.007

[2] Frye CA, Bo E, Calamandrei G, Calzà L, Dessì-Fulgheri F et al. (2012) Endocrine disrupters: a review of some sources, effects, and mechanisms of actions on behaviour and neuroendocrine systems. J Neuroendocrinol 24(1): 144-159. doi:10.1111/j.1365-2826.2011.02229.x. PubMed: 21951193. 21951193 10.1111/j.1365-2826.2011.02229.x PMC3245362

[3] Zhu BT (2002) Catechol-O-methyltransferase (COMT)-mediated methylation metabolism of endogenous bioactive catechols and modulation by endobiotics and xenobiotics: importance in pathophysiology and pathogenesis. Curr Drug Metab 3: 321–349. doi:10.2174/1389200023337586. PubMed: 12083324. 12083324 10.2174/1389200023337586

[4] Bonifácio MJ, Palma PN, Almeida L, Soares-da-Silva P (2007) Catechol-O-methyltransferase and its inhibitors in Parkinson’s disease. CNs Drugs Rev 13(3): 352-379. doi:10.1111/j.1527-3458.2007.00020.x. PubMed: 17894650. 10.1111/j.1527-3458.2007.00020.x PMC6494163 17894650

[5] Houston MC (2007) The role of mercury and cadmium heavy metals in vascular disease, hypertension, coronary heart disease, and myocardial infarction. Altern Ther Health Med 13(2): S128-S133. PubMed: 17405690. 17405690

[6] Lewandowski KE (2007) Relationship of catechol-O-methyltransferase to schizophrenia and its correlates: evidence for associations and complex interactions. Harv Rev Psychiatry 15(5): 233-244. doi:10.1080/10673220701650409. PubMed: 17924258. 17924258 10.1080/10673220701650409

[7] Tom T, Cummings JL (1998) Depression in Parkinson’s disease. Pharmacological characteristics and treatment. Drugs Aging 12(1): 55-74. doi:10.2165/00002512-199812010-00006. PubMed: 9467687. 9467687 10.2165/00002512-199812010-00006 PMC5786276

[8] Xie T, Ho SL, Ramsden DB (1999) Characterization and implications of estrogenic down-regulation of human catechol-O-methyltransferase gene transcription. Mol Pharmacol 56(1): 31-38. PubMed: 10385681. 10385681 10.1124/mol.56.1.31

[9] Yager JD, Liehr JG (1996) Molecular mechanisms of estrogen carcinogenesis. Annu Rev Pharmacol Toxicol 36: 203–232. doi:10.1146/annurev.pa.36.040196.001223. PubMed: 8725388. 8725388 10.1146/annurev.pa.36.040196.001223

[10] OECD (2001) Test No. 416: Two-Generation Reproduction Toxicity, OECD Guidelines for the Testing of Chemicals, Section 4. OECD Publishing doi:10.1787/9789264070868-en.

[11] Carvan MJ, Dalton TP, Stuart GW, Nebert DW (2000) Transgenic zebrafish as sentinels for aquatic pollution. Ann N Y Acad Sci 919: 133–147. PubMed: 11083105. 11083105 10.1111/j.1749-6632.2000.tb06875.x

[12] Soto AM, Sonnenschein C, Chung KL, Fernandez MF, Olea N et al. (1995) The E-SCREEN assay as a tool to identify estrogens: an update on estrogenic environmental pollutants. Environ Health Perspect 103 Suppl 7: 113-122. doi:10.2307/3432519. PubMed: 8593856. 10.1289/ehp.95103s7113 PMC1518887 8593856

[13] Jørgensen M, Vendelbo B, Skakkebaek NE, Leffers H (2000) Assaying estrogenicity by quantitating the expression levels of endogenous estrogen-regulated genes. Environ Health Perspect 108: 403–412. doi:10.1289/ehp.00108403. PubMed: 10811566. 10811566 10.1289/ehp.108-1638061 PMC1638061

[14] Soto AM, Maffini MV, Schaeberle CM, Sonnenschein C (2006) Strengths and weaknesses of in vitro assays for estrogenic and androgenic activity. Best Pract Res Clin Endocrinol Metab 20(1): 15-33. doi:10.1016/j.beem.2005.09.001. PubMed: 16522517. 16522517 10.1016/j.beem.2005.09.001

[15] Strunck E, Stemmann N, Hopert A, Wünsche W, Frank K, Vollmer G (2000) Relative binding affinity does not predict biological response to xenoestrogens in rat endometrial adenocarcinoma cells. J Steroid Biochem Mol Biol 74(3): 73-81. doi:10.1016/S0960-0760(00)00092-3. PubMed: 11086226. 11086226 10.1016/s0960-0760(00)00092-3

[16] Jiang H, Xie T, Ramsden DB, Ho SL (2003) Human catechol-O-methyltransferase down-regulation by estradiol. Neuropharmacology 45(7): 1011-1018. doi:10.1016/S0028-3908(03)00286-7. PubMed: 14573393. 14573393 10.1016/s0028-3908(03)00286-7

[17] Ho PWL, Chu ACY, Kwok KHH, Liu HF, Kung MHW et al. (2008) Effects of plasticisers and related compounds on the expression of the soluble form of catechol-O-methyltransferase in MCF-7 cells. Curr Drug Metab 9(4): 276-279. doi:10.2174/138920008784220628. PubMed: 18473745. 18473745 10.2174/138920008784220628

[18] Ho PWL, Garner CE, Ho JWM, Leung KC, Chu ACY et al. (2008) Estrogenic phenol and catechol metabolites of PCBs modulate catechol-O-methyltransferase expression via the estrogen receptor: potential contribution to cancer risk. Curr Drug Metab 9(4): 304-309. doi:10.2174/138920008784220600. PubMed: 18473748. 18473748 10.2174/138920008784220600

[19] Levenson AS, Jordan VC (1997) MCF-7: the first hormone-responsive breast cancer cell line. Cancer Res 57(15): 3071–3078. PubMed: 9242427. 9242427

[20] Melzer D, Harries L, Cipelli R, Henley W, Money C et al. (2011) Bisphenol A exposure is associated with in vivo estrogenic gene expression in adults. Environ Health Perspect 119(12): 1788-1793. doi:10.1289/ehp.1103809. PubMed: 21831745. 21831745 10.1289/ehp.1103809 PMC3261992

[21] Dawling S, Roodi N, Mernaugh RL, Wang X, Parl FF (2001) Catechol-O-methyltransferase (COMT)-mediated metabolism of catechol estrogens: comparison of wild-type and variant COMT isoforms. Cancer Res 61(18): 6716-6722. PubMed: 11559542. 11559542

[22] OECD (2011) Test No. 443: Extended One-Generation Reproductive Toxicity Study, OECD Guidelines for the Testing of Chemicals, Section 4. OECD Publishing doi:10.1787/9789264122550-en.

[23] Welshons WV, Nagel SC, Thayer KA, Judy BM, Vom Saal FS (1999) Low-dose bioactivity of xenoestrogens in animals: fetal exposure to low doses of methoxychlor and other xenoestrogens increases adult prostate size in mice. Toxicol Ind Health 15(1-2): 12-25. doi:10.1177/074823379901500103. PubMed: 10188188. 10188188 10.1177/074823379901500103

[24] Lawson C, Gieske M, Murdoch B, Ye P, Li Y et al. (2010) Gene Expression in the Fetal Mouse Ovary Is Altered by Exposure to Low Doses of Bisphenol A. Biol Reprod 84(1): 79-86. PubMed: 20739668. 20739668 10.1095/biolreprod.110.084814 PMC3012563

[25] Willhite CC, Ball GL, McLellan CJ (2008) Derivation of a bisphenol A oral reference dose (RfD) and drinking-water equivalent concentration. J Toxicol Environ Health B Crit Rev 11: 69–146. doi:10.1080/10937400701724303. PubMed: 18188738. 18188738 10.1080/10937400701724303

[26] Kim KH, Inan SY, Berman RF, Pessah IN (2009) Excitatory and inhibitory synaptic transmission is differentially influenced by two ortho-substituted polychlorinated biphenyls in the hippocampal slice preparation. Toxicol Appl Pharmacol 237(2): 168-177. doi:10.1016/j.taap.2009.03.002. PubMed: 19289137. 19289137 10.1016/j.taap.2009.03.002 PMC2693412

